# The Nipponbare genome and the next-generation of rice genomics research in Japan

**DOI:** 10.1186/s12284-016-0107-4

**Published:** 2016-07-22

**Authors:** Takashi Matsumoto, Jianzhong Wu, Takeshi Itoh, Hisataka Numa, Baltazar Antonio, Takuji Sasaki

**Affiliations:** National Institute of Agrobiological Sciences, 2-1-2 Kannondai, Tsukuba, Ibaraki 305-8602 Japan; Present Address: National Agriculture and Food Research Organization, 2-1-2 Kannondai, Tsukuba, Ibaraki 305-8518 Japan; Nodai Research Institute, Tokyo University of Agriculture, 1-1-1 Sakuragaoka, Setagaya, Tokyo, 156-8502 Japan

**Keywords:** Rice, *Oryza sativa*, Nipponbare, Genome, Annotation, Transcriptome, Agronomic traits

## Abstract

The map-based genome sequence of the *japonica* rice cultivar Nipponbare remains to date as the only monocot genome that has been sequenced to a high-quality level. It has become the reference sequence for understanding the diversity among thousands of rice cultivars and its wild relatives as well as the major cereal crops that comprised the food source for the entire human race. This review focuses on the accomplishments in rice genomics in Japan encompassing the last 10 years which have led into deeper understanding of the genome, characterization of many agronomic traits, comprehensive analysis of the transcriptome, and the map-based cloning of many genes associated with agronomic traits.

**Electronic supplementary material:**

The online version of this article (doi:10.1186/s12284-016-0107-4) contains supplementary material, which is available to authorized users.

## Introduction

The elucidation of the rice genome sequence is a major milestone in science as it paves the way for understanding the biology of a major cereal crop that feeds more than half of the world’s population (International Rice Genome Sequencing Project 2005). Although roughly a hundred plant genome sequences have already been published to date, the map-based sequence of *Oryza sativa* ssp. *japonica* cv. Nipponbare remains as the only monocot genome that has been sequenced to a high-quality level. It has therefore become a reference for sequencing of other cereal crops with much larger genome sizes such as maize (Schnable et al. 2009), sorghum (Paterson et al. 2009), soybean (Schmutz et al. 2010), barley (International Barley Genome Sequencing Consortium 2012), and wheat (International Wheat Genome Sequencing Consortium 2014). More importantly, the rice genome sequence has become the most powerful tool in agriculture enhancing the ability of breeders to develop new cultivars with highly desirable traits such as high yield, resistance to biotic/abiotic stress, good eating quality, and cultivars that could adapt to an ever changing cultivation environment brought about by global warming. It is expected that subsequent sequencing of a wide array of rice germplasm throughout the world will be the platform for propelling the next green revolution to increase productivity under more sustainable conditions.

Although 90 % of rice is consumed mainly in Asia, it is also a major food source in many African and South American countries. Rice is a main staple in the Japanese diet with the current average per capita consumption of about 60 kg per year. It has been cultivated both as a staple and economic crop for more than 2000 years across the country and has been integrated in many aspects of the culture as well. Thousands of cultivars have been developed as a result of crossbreeding and selection conducted by farmers and breeders to suit the specific local conditions. Therefore, the complete rice genome sequence based on the cultivar Nipponbare led to the large-scale characterization of other *japonica* cultivars including the widely cultivated and elite cultivar Koshihikari (Yamamoto et al. 2010) known for good eating quality.

This review will focus on the accomplishments in rice genomics in Japan encompassing the last 10 years since the completion of the rice genome sequence. There is no doubt however that a great deal of accomplishments has been achieved not only by the 10 participating countries in the international sequencing consortium but also by many rice researchers worldwide who have continuously engaged in understanding the rice biology based on the map-based Nipponbare genome sequence. In Japan, succeeding efforts in genome analysis from 2005 onwards have led to fine tuning of the genome assembly, deeper understanding of the structure of specific regions of the genome, characterization of many important traits across various cultivars, comprehensive profiling of the transcriptome, and the isolation and map-based cloning of many genes associated with agronomic traits.

## Review

### Enhancing the genome assembly and annotation

**Fig. 1:**
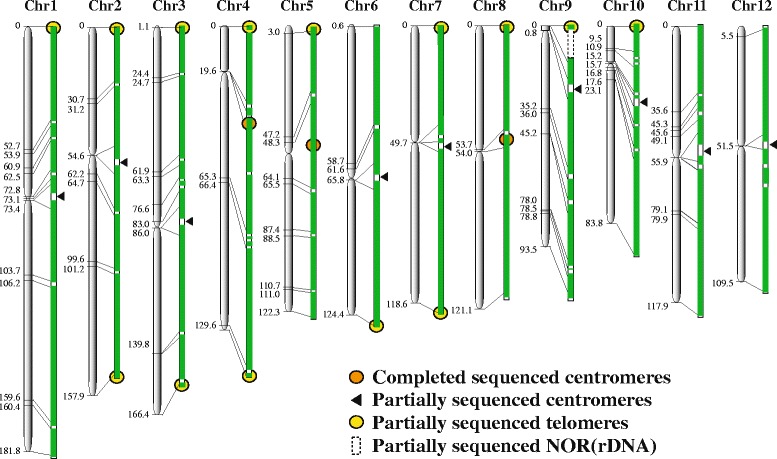
Current status of the Nipponbare pseudomolecules. The coverage of the genome sequence for each chromosome indicated as green bars is shown with the corresponding genetic map distance (cM). The remaining gaps indicated as white areas include several centromeres, telomeres and a few regions in each chromosome

There have been continuous efforts to refine the genome assembly and enhance the annotation of the genes since the publication of the high-quality map-based sequence of the *japonica* cultivar Nipponbare. These efforts focused on gap-filling of the 12 chromosomes and characterization of the complex regions of the genome such as the centromeres, telomeres and nucleolar-organizing regions. Among the 12 chromosomes, the complex and highly repetitive centromere-specific DNA sequences were first reported in *Cen4* (Zhang et al. 2004), *Cen8* (Nagaki et al. 2004; Wu et al. 2004), and subsequently *Cen3* (Yan et al. 2006) which also complemented the previous extensive works on rice centromeres (Jiang et al. 1996; Cheng et al. 2002). We have continued to improve the quality of the Nipponbare genome pseudomolecules even after the completion of the IRGSP sequencing initiative. Using BAC sequence analysis, genome annotation, and FISH analysis, we characterized the nearly completed and high-quality genomic sequence of *Cen5* in chromosome 5 and revealed some striking differences among the centromeres in terms of the copy number and distribution pattern of the centromere-specific satellite repeat CentO as well as the distribution and expression of transcription units within the pericentromeric and centromeric regions (Mizuno et al. 2011). In the case of the telomeres, Fibre-FISH analysis revealed the presence of arrays of 730–1500 conserved copies of telomere-specific 5’-TTTAGGG-3’ repeat sequence at the end regions of chromosomes 1S, 2S, 2L, 6L, 7S, 7L and 8S of Nipponbare (Mizuno et al. 2006). Gene annotation from the 500 kb subtelomere sequences clearly indicated that the rice chromosomal ends were gene-rich with high transcriptional expression. In addition, the subtelomere regions on these chromosome ends hardly contained TrsA, a subtelomeric repeat sequence of rice. On the other hand, clusters of TrsA have been observed in chromosomes 5L, 6S, 8L, 9L and 12L (Mizuno et al. 2008a). Sequence comparison of these 14 telomere-ends and telomere-flanking regions also revealed the occurrence of deletions, insertions, or chromosome-specific substitutions of single nucleotides within the telomere specific repeats at the junction between the telomere and subtelomere, suggesting the telomeric variants in rice have arisen from the rapid expansion of a single mutation rather than from the gradual accumulation of random mutations (Mizuno et al. 2008b). More recently, the 14 telomere-ends from 12 chromosomes were successfully constructed from a fosmid library leading to the identification of telomere sequences and structure in rice (Mizuno et al. 2014). These additional sequenced regions of the genome have been incorporated into the genome assembly as we update the pseudomolecules on a regular basis. The most recent physical map of the genome covers almost 97 % of the entire genome with 62 remaining physical gaps (Fig. 1).

The latest genome assembly was constructed as a joint effort of the Rice Annotation Project Database (RAP-DB) of the National Institute of Agrobiological Sciences (NIAS) and the Michigan State University (MSU) Rice Genome Annotation Project to update and validate the reference IRGSP Nipponbare genome sequence and provide a unified set of pseudomolecules to the rice research community (Kawahara et al. 2013). The genome assembly was revised using the rice optical map (Zhou et al. 2007) to validate the minimal tiling path. The next-generation sequencing (NGS) data obtained by re-sequencing two individual Nipponbare plants using the Illumina Genome Analyzer II/IIx and Roche 454 GS FLX were used to check sequencing errors in the revised assembly. This resulted in the identification of 4886 sequencing errors and five insertions/deletions in the 321 Mb of the assembled genome corresponding to an error rate of 0.15 per 10,000 nucleotides in the original IRGSP assembly. The revised and unified genome assembly, Os-Nipponbare-Reference-IRGSP-1.0 (IRGSP-1.0), is now used to provide a common platform for genome annotation in the RAP-DB (http://rapdb.dna.affrc.go.jp, Rice Annotation Project 2008) and the MSU rice annotation database (http://rice.plantbiology.msu.edu/cgi-bin/gbrowse/rice/).

In line with the revision of the genome assembly, the RAP-DB has been enhanced further with the mapping of 154,579 transcript sequences from the genus *Oryza* and other monocot species (Sakai et al. 2013). In addition, literature-based manually curated data, transcriptome data, and NGS data of major rice cultivars were also incorporated into the database. The current release of RAP-DB consists of 37,869 loci including 1626 loci that correspond to literature-based manually curated annotation data, commonly used gene names, and gene symbols. Transcription data derived from Illumina RNA-seq analysis of various tissues under normal and stress conditions (Mizuno et al. 2010; Oono et al. 2011; Kawahara et al. 2012, 2016) have been added to enhance the utility of the database in understanding transcriptional regulatory networks. Links to gene families in rice, *Sorghum bicolor*, *Zea mays* and *Arabidopsis thaliana* are provided to facilitate analysis of how genes are conserved and evolved among plant species. An additional feature to RAP-DB is the Short-Read Assembly Browser (S-RAB) that provides a viewer for Illumina reads of the *japonica* cultivar Koshihikari and *indica* cultivar Guangluai-4 mapped to the Nipponbare genome, showing the alignments, single nucleotide polymorphisms (SNPs), and gene functional annotations. The RAP-DB is updated on a regular basis so that it can provide researchers with the latest information on characterization of rice genome structure and function. Recent advances in DNA sequencing technologies resulted in generation of massive genome sequencing data in a considerable number of rice cultivars and species. To facilitate efficient visualization of rapidly emerging large-scale sequencing data, a novel web-based browser, Tasuke (http://tasuke.dna.affrc.go.jp/), with various functions to show the variation and read depth of multiple genomes, as well as annotations and SNP data of hundreds of cultivars aligned to a reference genome at various scales, has been developed for efficient utilization of emerging NGS data of rice cultivars (Kumagai et al. 2013).

### Deeper perspectives on rice genetic resources

The reference Nipponbare genome facilitated the sequencing initiatives of rice germplasm aimed at understanding the genetic diversity which led to the domestication of rice as grown today. Foremost among these initiatives are the Oryza Map Alignment Project (OMAP) to clarify the diversity in the twelve wild rice genomes (Wing et al. 2007), and the international effort of resequencing a core collection of 3000 rice accessions from 89 countries to provide a foundation for large-scale discovery of novel alleles for important rice phenotypes (The 3,000 Rice Genomes Project 2014; Huang et al. 2012). To facilitate comprehensive understanding of the genome diversity in rice, we have also sequenced the African rice *O. glaberrima* known to be more resilient to water shortage as well as fungal or insect diseases than *O. sativa* (Sakai et al. 2011). The high-quality assembly and annotation of the *O. glaberrima* genome have also been reported, providing evidence for its independent domestication (Wang et al. 2014).

In contrast, successive efforts in Japan focus on understanding cultivars important to Japanese agriculture particularly those widely grown throughout Japan. To date, whole genome sequences from 16 varieties obtained by next-generation sequencers have been submitted in public databases by NIAS and other Japanese research organizations (Additional file 1: Table S2), and projects for sequencing other varieties and landraces are in progress.

The cultivar Koshihikari developed in 1953 is the most widely grown and favored cultivar in Japan occupying almost 80 % of total rice production including its relative cultivars. Many breeding efforts focus on further improvement of quality depending on the region where it is grown. The genome sequence of Koshihikari is therefore indispensable in breeding and designing rice to meet the demands of Japanese consumers. With the reference Nipponbare sequence, the next development was the sequencing of the Koshihikari genome with the Illumina sequencing technology (Yamamoto et al. 2010). A total of 67,051 SNPs between Koshihikari and Nipponbare, some of which derived from originating landraces and distributed through Koshihikari relatives, and 18 pedigree haplotype blocks which were artificially selected during breeding.

The Nipponbare pseudomolecule sequence was used as the template in the construction of a complete BAC-based physical map of the *O. sativa* ssp. *indica* cv. Kasalath (Kanamori et al. 2013). We also sequenced the centromere region of chromosome 8 in Kasalath (Wu et al. 2009). Comparative analysis with Nipponbare Cen8 revealed both collinearity and diversity in each orthologous centromere. Subsequently, deep sequencing (>154-fold coverage) via the Roche GS-FLX Titanium or GS-FLX+ and Illumina GAIIx or HiSeq 2000 platforms and *de novo* assembly generated the 330.55 Mb Kasalath pseudomolecule sequence representing 91.1 % of the genome with 35,139 expressed loci annotated by RNA-Seq analysis (Sakai et al. 2014). Comparison of the Kasalath pseudomolecule with Nipponbare revealed 2,787,250 SNPs and 7393 large indel sites (>100 bp). On the other hand, comparison with the *indica* cultivar 93–11 showed 2,216,251 SNPs and 3780 large indels (Sakai et al. 2014). In particular, at least 14.78 Mb of indel sequences and 40.75 Mb of unmapped sequences were identified in the Kasalath genome in comparison with the Nipponbare genome suggesting that ~6.3 % of the total transcript loci in rice genome is presumably involved with gain or loss of genes.

Genotyping of the NIAS Genebank (https://www.gene.affrc.go.jp/index_en.php) rice accessions with 179 RFLP markers led to development of a rice diversity research set of germplasms (RDRS) in *indica*, *aus* and *japonica* accessions available for the detailed genetic studies and rice improvement (Kojima et al. 2005). Based on a result from screening 234 accessions of rice collected in Asia, the Americas, Africa, Europe and Oceania with 169 SSR (simple sequence repeats) markers, moreover, current *O. sativa* cultivars and landraces can be classified in more detail into five genetically differentiated groups: *indica*, *aus*, *aromatic*, *temperate japonica*, and *tropical japonica* because of its deep genetic structure evolved during domestication and adaptation and its autogamous breeding system (Garris et al. 2005).

**Fig. 2:**
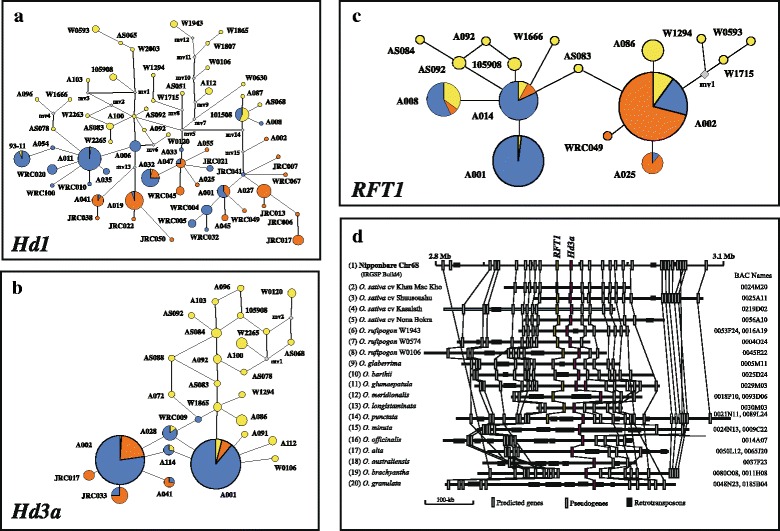
Molecular and evolutionary analysis of *flowering* genes across rice accessions. The major flowering genes, namely, *Hd1*, *Hd3a* and *RFT1* genes were analysed using the exon sequences of 202 rice accessions. A-C: The resulting haplotype network of *Hd1* (**a**), *Hd3a* (**b**) and *RFT1* (**c**) constructed with the corresponding accession name and size proportional to the total number of samples from *O. rufipogon* (*yellow*), *O. sativa* ssp. *indica* (*blue*) and *O. sativa* ssp. *japonica* (*orange*). Lines between haplotypes represent the mutational steps between alleles. Hypothetical haplotypes (median vector) with discontinuous links are indicated as grey squares. **d**: Compositional and structural comparison of the *Hd3a* (*red box*), and *RFT1* region (*orange box*) across various Oryza species. The genes with loss-of-function (*white box*) and retrotransposons (*black box*) are also shown. Orthologous genes are linked by solid lines. The BAC sequences correspond to DDBJ accessions AP011450 ~ AP011476

A series of comparative genomic studies among various species in the genus *Oryza* focused on a number of domestication or adaptation related genes such as the *sh4* gene region responsible for the reduction of grain shattering, the semi-dwarf1 (*sd-1*) gene (Wu et al. 2008; Asano et al. 2011), the major heading-date related genes such as *Hd1*, *Hd3a*, *Hd6*, *RFT1* and *Ghd7* (Fujino et al. 2010; Yamane et al. 2009; Ebana et al. 2011). Analysis of expression levels revealed clear association of the functional and nonfunctional alleles with early and late flowering, suggesting that *Hd1* is a major determinant of variation in flowering time of cultivated rice (Takahashi et al. 2009). Sequencing of BAC clones covering the chromosomal region of *Hd3a* and *RFT1* genes across the AA ~ GG genomes revealed that at least 89 % of the amino acid sequences encoded by *Hd3a* which promotes the transition to flowering under the short-day condition were conserved across the different *Oryza* species (Komiya et al. 2008). In comparison with *Hd1*, the *Hd3a* gene obviously showed much less genetic diversity with 95 ~ 100 % sequence identity among the accessions of *O. sativa* and *O. rufipogon* (Fig. 2a, b). Similarly, the *RFT1* gene which has been associated with late flowering of rice under long-day condition in a functional *Hd1* background (Ogiso-Tanaka et al. 2013) also showed low genetic diversity (Fig. 2c). Extremely high gene collinearity was also found in the surrounding region (~300kbp) of *Hd3a* and *RFT1* genes across the *Oryza* species despite the size differences caused mainly by transposable element insertions (Fig. 2d). Unlike *Hd3a*, the *RFT1* gene has only been found in the *Oryza* species that have the AA (including *O. sativa*) or BB genomes (*O. punctata*). These genomes diverged from a common ancestor only ~2 Mya. This result suggests that the *RFT1* gene may have originated from *Hd3a* by a recent duplication although a possible deletion of *RFT1* within the other species could not be ruled out. The Nipponbare reference sequence could contribute insights into the molecular mechanisms underlying genomic evolution and selection in rice which would benefit breeding programs to modify and control flowering time through efficient utilization of different genes or gene alleles.

### Elucidating the molecular function of rice genes

The rice genome sequence of Nipponbare has been pivotal in the development of a system for discovering the biological functions of approximately 32,000 genes identified in rice. This has been addressed early on with the development of resources for functional genomics such as the *Tos17* insertion mutant panel (Hirochika 2001) and the rice full-length cDNA collection (Kikuchi et al. 2003). The Tos17 mutant collection consisting of approximately 50,000 mutant lines characterized into flanking sequences have been used in full characterization of many genes characterized in Japan and nearly 50 *Tos17* mutant lines have been successfully used in tagging specific genes (See reference list at https://tos.nias.affrc.go.jp/doc/references.html).

Similarly, the information from approximately 28,000 fully-sequenced cDNA clones and the KOME Database (hhttps://dbarchive.biosciencedbc.jp/en/kome/desc.html) have been very useful for functional characterization of many genes. Both these resources are still widely used up to the present by researchers around the world particularly in systematically assigning functions to many predicted genes in the genome, in addition to other resources such as T-DNA insertion lines (Jung and An 2013), Ac/Ds tagging lines (Guiderdoni and Gantet 2012), nDART/aDart lines (Takagi et al. 2007). Analysis of the flanking sequences of these insertional mutants resulted in accumulation of nearly 448,000 gene-tagging sequence resources for characterization of gene functions (Wei et al. 2013).

**Fig. 3:**
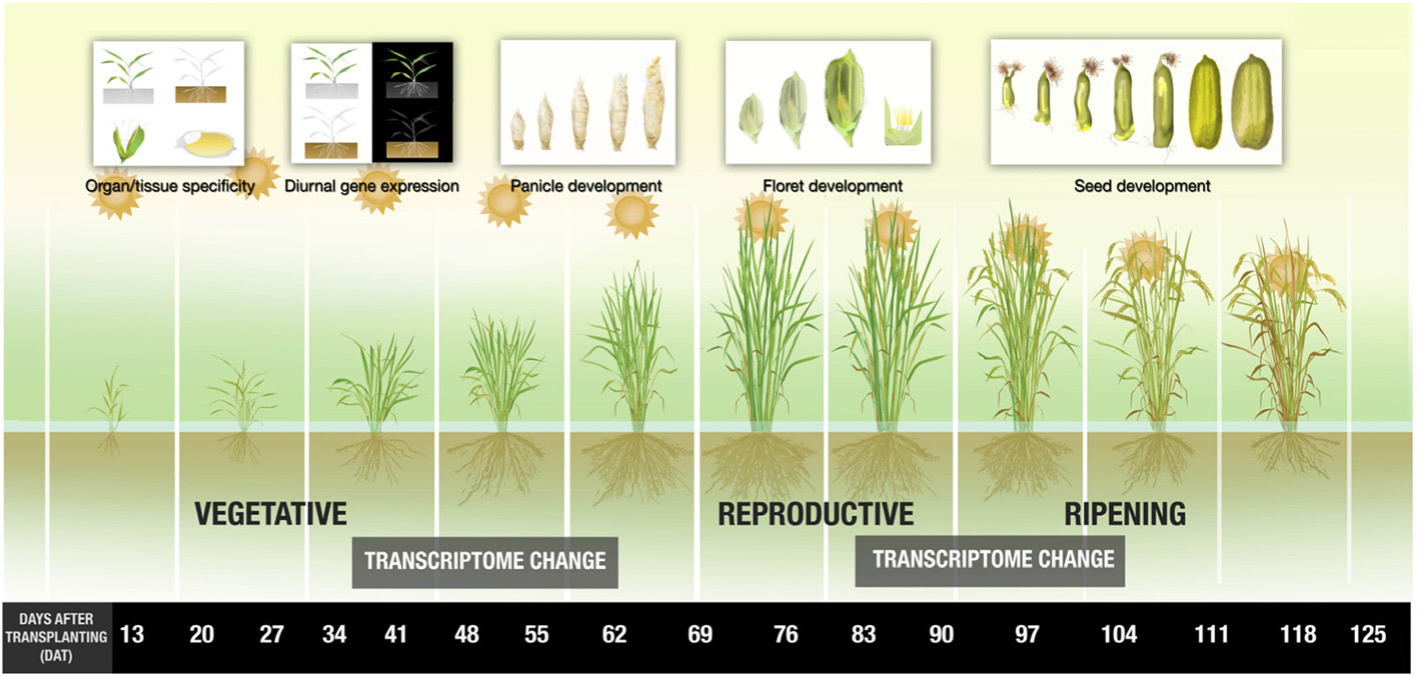
Characterizing the field transcriptome of rice. Microarray analysis was used to characterize gene expression of rice cells and tissues at various stages of development from transplanting to harvesting in the field

The rice full-length cDNAs also serve as the main resources for large-scale gene expression profiling. Using these sequences as well as predicted gene information for plausible probes, we have designed 44 K Agilent oligonucleotide microarray to analyze the field transcriptome of field-grown rice (Fig. 3). A wide range of gene expression profiles based on organs and tissues at various developmental stages identified organ/tissue specific genes as well as growth stage-specific genes. Continuous transcriptome profiling of leaf from transplanting until harvesting stage uncovered two major drastic changes in the leaf transcriptional program (Sato et al. 2011). The rice transcriptome is well documented in two databases, namely, RiceXPro (http://ricexpro.dna.affrc.go.jp/) providing an overview of the transcriptional changes throughout the growth of the rice plant in the field, and RiceFREND (http://ricefrend.dna.affrc.go.jp/) for co-expression analysis of these genes. These resources are now widely used for deciphering gene functions and analysis of rice gene networks. Combining the massive field transcriptomic data and meteorological information with statistical model construction, we have also succeeded, to some extent, in predicting fluctuation of gene expression, or transcriptome dynamics (Nagano et al. 2012). These predictions may give insights for improving crop production, disease resistance and resilience to global stress. Information from transcription profiles can also predict the best cultivation conditions for a given variety in a given location.

With current advances in next-generation sequencing, we have embarked on analysis of gene expression using RNA-seq to measure the presence and quantity of RNA transcripts under specific growth conditions. As a result, unannotated salinity stress-inducible transcripts have been identified using the RNA-seq profile of seedlings treated with NaCl (Mizuno et al. 2010). Through RNA-seq analysis, previously unannotated salinity response genes, some of which might function in phosphate and cadmium stress tolerance were discovered. Simultaneous measurement of rice and rice blast fungus transcripts in infected plants using RNA-seq provided infection-responsive expression profiles for both rice and fungal transcripts. These profiles might indicate genes that may interact at different times and different tissues during infection (Kawahara et al. 2012). The same strategy has been used to characterize the transcriptome profile of *japonica* cultivar Nipponbare under phosphate starvation (Oono et al. 2011, 2013), as well as the diversity among the transcriptomes of rice cultivars including Nipponbare with low tolerance to phosphate starvation stress, *japonica* cultivars IAC 25 and Vary Lava 701 with relatively higher tolerance, and *indica* cultivar Kasalath known to be highly tolerant to phosphate stress (Oono et al. 2013). Recently, we have also found that cadmium stress controls the expression of genes in drought stress signal pathways in rice based on genome wide transcriptome analysis (Oono et al. 2014).

### Isolation and utilization of agronomically important genes

The genome sequence coupled with genomics tools and resources such as mutant lines, genetic populations and germplasm collection paved the way for marker-assisted selection, mapping of QTLs and map-based cloning of specific genes having agronomic properties. As a result, many QTLs have been detected as Mendelian factors in rice, including those responsible for increased yield, resistance to various insect pests and diseases, resistance to abiotic stress such as drought, salinity and submergence, good eating quality etc.

#### Heading date

Many QTLs involved in heading date, a key determinant of rice adaptation to different cultivation areas and cropping seasons, have been characterized. These include *Hd1*, *Hd2*, *Hd3a*, *Hd3b*, *Hd4*, *Hd5*, *Hd6*, *Hd8*, and *Hd9* (Yano et al. 2001). Subsequent studies focused on more detailed characterization of these QTLs based on the genome sequence. The *Hd1* contains a CCT domain with ~60 % identity to *Ghd7*, a long day dependent negative regulator of heading date. *Hd6* encodes casein kinase 2 alpha, and *Hd3a* is similar to an *Arabidopsis* FT-like protein (Takahashi et al. 2001; Kojima et al. 2002). Another major QTL, *Early heading date 1* (*Ehd1*), encodes a B-type response regulator that is suppressed under long-day conditions for which *Ghd7* is responsible (Doi et al. 2004). In more recent studies, it has been found that *Ehd3*, encoding a PHD finger-containing protein, is a critical promoter of rice flowering (Matsubara et al. 2011). *Hd17,* a homolog of Arabidopsis *EARLY FLOWERING 3* (*ELF3)*, is involved in the photoperiodic flowering pathway by regulating the transcription level of a flowering repressor, *Grain number, plant height and heading date 7* (*Ghd7*) gene (Matsubara et al. 2012). The QTL *Hd16* is a gene for casein kinase I which is involved in the control of rice flowering time by modulating the day-length response (Hori et al. 2013).

#### Disease resistance

Developing cultivars with broad spectrum of resistance to diseases is a priority for rice breeding in Japan. Among the genes widely characterized for disease resistance, the rice *WRKY4*5 gene has been found to play a crucial role in resistance to bacterial and fungal blast induced by benzothiadiazole (BTH), a so-called plant activator that protects plants from diseases by activating plant innate immune system (Shimono et al. 2007). The *Pib* gene is the first cloned gene for resistance to blast induced by *Magnaporthe oryzae* (Wang et al. 1999) and it was reported much later that *Pi21* encodes a proline-rich protein with a heavy metal-binding domain and putative protein-protein interaction motifs (Fukuoka et al. 2009).

#### Domestication

The Nipponbare genome sequence has been instrumental in deciphering the evolutionary processes in the domestication of rice (Yang et al. 2012). Several genes that play key roles in selection and domestication have been analyzed. The seed shattering *qSH1* gene has been found to encode a BEL1-type homeobox gene and a SNP in the 5’ regulatory region caused a loss of seed shattering owing to the absence of abscission layer formation (Konishi et al. 2006). More recently, *Kala4,* the gene responsible for the black color of rice grains (also referred to as purple rice) has been identified based on an extensive analysis of the genes associated with grain color in about 50 rice cultivars, tracing the origin to tropical *japonica* (Oikawa et al. 2015).

#### Abiotic tolerance

Map-based cloning of *qLTG3-1* which controls low-temperature germination in rice provides useful insights on cultivation in temperate as well as high altitude rice growing areas (Fujino et al. 2008). The molecular mechanism of deepwater response has been clarified through the identification of the genes *SNORKEL1* and *SNORKEL2*, which trigger deepwater response by encoding ethylene response factors involved in ethylene signaling (Hattori et al. 2009). With the molecular cloning of *Sdr4*, a seed dormancy QTL in rice, the role of the gene as an intermediate regulator of dormancy in the seed maturation program has been clarified (Sugimoto et al. 2010). The *DEEPER ROOTING 1* (*Dro1*) has been recently discovered through screening and genetic analysis of the NIAS rice collection which have a great potential for improvement of rice yield under drought conditions by controlling the root system architecture in rice (Uga et al. 2013).

#### Yield

The major components that determine yield in rice have been widely characterized using the sequence information. The *qSW5* gene which corresponds to the QTL for seed width on chromosome 5 has been cloned and a deletion in the gene was found to be associated with larger grain size (Shomura et al. 2008). Furthermore, it has also been shown that this variant was selected during rice domestication for increased yields. Characterization of genes associated with productivity has also made significant progress. A loss-of-function mutation of rice *DENSE PANICLE 1* causes semi-dwarfness and slightly increased number of spikelets (Taguchi-Shiobara et al. 2011). A natural variant of *NARROW LEAF 1* (*NAL1*) gene selected in high-yield rice breeding programs increased the photosynthesis rate (Takai et al. 2013; Fujita et al. 2013). The *THOUSAND-GRAIN WEIGHT 6* (*TGW6*) gene limits endosperm cell number and grain length. Defective alleles lead to increase in grain size and yield (Ishimaru et al. 2013). Also researchers at Nagoya U. revealed the basic molecular strategy for construction good plant type for ideal yield performance, most of which related to metabolism or biosynthesis of plant hormones (Ueguchi-Tanaka et al. 2005; Ikeda et al. 2013)

**Fig. 4:**
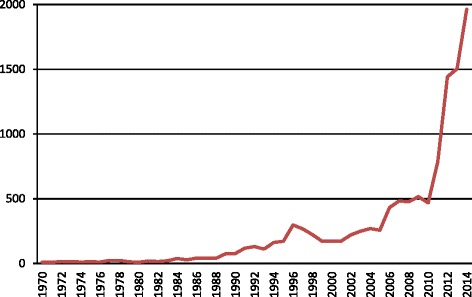
The growth of rice publications before and after the completion of the rice genome sequence. The number of publications on rice research from 1970 to 2014 showed significant increase after the completion of the Nipponbare genome sequence in 2004

A list of genes that have been characterized mainly or in collaboration with Japanese researchers in 2005–2014 is summarized in Additional file 2: Table S1. Significant contributions have been made in elucidating gene functions, identifying QTLs, characterizing molecular mechanisms, and establishing the DNA marker-assisted selection (MAS) as a precise and effective breeding strategy to produce novel varieties. There is no doubt that in the last 10 years since the completion of the rice genome, worldwide rice research has made significant output as evidenced in the number of rice related publications. An overview of the trend in rice research in the last 45 years is shown in Fig. 4 (data provided by Oryzabase, Kurata and Yamazaki 2006). Since 2005, the number of publications doubled in just a matter of 5 years (2010) and by the end of 2014, there are almost 2000 publications on rice alone (including 67 from Japan). In total, Japanese researcher contributed about 70–100 per year in the last 10 years since the completion of the rice genome sequence in 2004.

## Conclusion

In Japan, rice genomics has been a part of major research programs of the Ministry of Agriculture, Forestry and Fisheries (MAFF) that address various issues in sustainable food production and agriculture. Foremost among these issues are the rapid aging of farm workers and depopulation of farming communities which would eventually affect agricultural production in the not so distant future. In breeding programs, various efforts are being initiated to integrate rice genomics technology to the development of novel varieties to reinvigorate Japanese agriculture. The new basic plan for food, agriculture and the rural areas which serve as the guideline for advancing the reform of measures and efforts by the entire nation so as to enable Japan’s agriculture and rural areas to accurately respond to structural and other changes in the economy and society (http://www.maff.go.jp/e/basic_law/basiclaw_agri/basiclaw_agri.html). Rice being an integral part of Japanese agriculture is very much a part of these programs. Several ongoing MAFF-funded research projects focus on using the genome information of various crops for the development of various technologies to boost next-generation agriculture. As a part of that major project, the development of rice genomics resources and informatics tools are expected to contribute in such areas through attempts of various sectors to develop rice cultivars that address the specific needs of various rice producing regions in Japan considering the environmental changes in rice cultivation due to climate change.

As the rice research community embarks on various efforts in rice genomics, more rice genome sequence and transcriptome profiles will be generated in the very near future. Elucidating the molecular mechanisms controlling many biological processes will be supplemented by information to be obtained from other technologies such as proteomics, metabolomics (Okazaki and Saito 2016), epigenomics (Chen and Zhou 2013), and phenomics (Yang et al. 2013). Integration of all these data via advanced bioinformatics will elucidate the gene cascade or network in the whole rice plant that will serve as the platform on how to utilize and improve crop function. And Japanese researchers will continue to be a part of various initiatives for these advancements will most likely revolutionize rice breeding to circumvent future concerns of sustainable agriculture and food security.

## Abbreviations

IRGSP, International Rice Genome Sequencing Project; NGS, next-generation sequencing technology; RAP-DB, Rice Genome Annotation Project Database

## Additional files

Additional file 1: Table S2. Rice varieties and landraces with whole-genome short-read sequences submitted by Japanese research organizations in the NCBI database. (XLSX 20 kb) Additional file 2: Table S1. Rice genes reported by Japanese researchers in various scientific journals in 2005–2014. Literatures were searched and obtained from PubMed with ‘rice’ and ‘Oryza’ as keywords in either the title or abstract, and further selected by natural language processing and manual curation. The data can be accessed from Oryzabase (http://shigen.nig.ac.jp/rice/oryzabase/download/reference). (XLSX 215 kb)

## References

[CR1] Asano K, Yamasaki M, Takuno S, Miura K, Katagiri S, Ito T, Doi K, Wu J, Ebana K, Matsumoto T, Innan H, Kitano H, Ashikari M, Matsuoka M (2011). Artificial selection for a green revolution gene during *japonica* rice domestication. Proc Natl Acad Sci U S A.

[CR2] Chen X, Zhou D-X (2013). Rice epigenomics and epigenetics: challenges and opportunities. Curr Opin Plant Biol.

[CR3] Cheng Z, Dong F, Langdon T, Ouyang S, Buell R, Gu M, Blattner F, Jiang J (2002). Functional rice centromeres are marked by a satellite repeat and a centromere-specific retrotransposon. Plant Cell.

[CR4] Doi K, Izawa T, Fuse T, Yamanouchi U (2004). *Ehd1*, a B-type response regulator in rice, confers short-day promotion of flowering and controls FT-like gene expression independently of *Hd1*. Genes Dev.

[CR5] Ebana K, Shibaya T, Wu J, Matsubara K, Kanamori H, Yamane H, Yamanouchi U, Mizubayashi T, Kono I, Shomura A, Ito S, Ando T, Hori K, Matsumoto T, Yano M (2011). Uncovering of major genetic factors generating naturally occurring variation in heading date among Asian rice cultivars. Theor Appl Genet.

[CR6] Fujino K, Sekiguchi H, Matsuda Y, Sugimoto K, Ono K, Yano M (2008). Molecular identification of a major quantitative trait locus, *qLTG3-1*, controlling low-temperature germinability in rice. Proc Natl Acad Sci U S A.

[CR7] Fujino K, Wu J, Sekiguchi H, Ito T, Izawa T, Matsumoto T (2010). Multiple introgression events surrounding the *Hd1* flowering-time gene in cultivated rice, *Oryza sativa* L. Mol Genet Genomics.

[CR8] Fujita D, Trijatmiko KR, Tagle AJ, Sapasap MV, Koide Y, Sasaki K, Tsakirpaloglou N, Gannaban RB, Nishimura T, Yanagihara S, Fukuta Y, Koshiba T, Slamet-Loedin IHS, Ishimaru T, Kobayashi N (2013). *NAL1* allele from a rice landrace greatly increase yield in modern *indica* cultivars. Proc Natl Acad Sci U S A.

[CR9] Fukuoka S, Saka N, Koga H, Ono K, Shimizu T, Ebana K, Hayashi N, Takahashi A, Hirochika H, Okuno K, Yano M (2009). Loss of function of a proline-containing protein confers durable disease resistance in rice. Science.

[CR10] Garris AJ, Tai TH, Coburn J, Kresovich S, McCouch S (2005). Genetic structure and diversity in *Oryza sativa* L. Genetics.

[CR11] Guiderdoni E, Gantet P (2012). Ac-Ds solutions for rice insertion mutagenesis. Methods Mol Biol.

[CR12] Hattori Y, Nagai K, Furukawa S, Song X, Kawano R, Sakakibara H, Wu J, Matsumoto T, Yoshimura A, Kitano H, Matsuoka M, Mori H, Ashikari M (2009). The ethylene response factors *SNORKEL1* and *SNORKEL2* allow rice to adapt to deep water. Nature.

[CR13] Hirochika H (2001). Contribution of the Tos17 retrotransposon to rice functional genomics. Curr Opin Plant Biol.

[CR14] Hori K, Ogiso-Tanaka E, Matsubara K, Yamanouchi U, Ebana K, Yano M (2013). *Hd16*, a gene for casein kinase I, is involved in the control of rice flowering time by modulating the day-length response. Plant J.

[CR15] Huang X, Kurata N, Wei X, Wang ZX, Wang A, Zhao Q, Zhao Y, Liu K, Lu H, Li W, Guo Y, Lu Y, Zhou C, Fan D, Weng Q, Zhu C, Huang T, Zhang L, Wang Y, Feng L, Furuumi H, Kubo T, Miyabayashi T, Yuan X, Xu Q, Dong G, Zhan Q, Li C, Fujiyama A, Toyoda A, Lu T, Feng Q, Qian Q, Li J, Han B (2012). A map of rice genome variation reveals the origin of cultivated rice. Nature.

[CR16] Ikeda M, Miura K, Aya K, Kitano H, Matsuoka M (2013). Genes offering the potential for designing yield-related traits in rice. Curr Opin Plant Biol.

[CR17] International Barley Genome Sequencing Consortium (2012). A physical, genetic and functional sequence assembly of the barley genome. Nature.

[CR18] International Rice Genome Sequencing Project (2005). The map-based sequence of the rice genome. Nature.

[CR19] International Wheat Genome Sequencing Consortium (2014). A chromosome-based draft sequence of the hexaploid bread wheat (*Triticum aestivum*) genome. Science.

[CR20] Ishimaru K, Hirotsu N, Madoka Y, Murakami N, Hara N, Onodera H, Kashiwagi T, Ujiie K, Shimizu B, Onishi A, Miyagawa H, Katoh E (2013). Loss of function of the IAA-glucose hydrolase gene *TGW6* enhances rice grain weight and increases yield. Nat Genet.

[CR21] Jiang J, Nasuda S, Dong F, Scherrer CW, Woo SS, Wing RA, Gill BS, Ward DC (1996). A conserved repetitive DNA element located in the centromeres of cereal chromosomes. Proc. Natl Acad Sci USA.

[CR22] Jung KH, An G (2013). Functional characterization of rice genes using a gene-indexed T-DNA insertional mutant population. Methods Mol Biol.

[CR23] Kanamori H, Fujisawa M, Katagiri S, Oono Y, Fujisawa H, Karasawa W, Kurita K, Sasaki H, Mori S, Hamada M, Mukai Y, Yazawa T, Mizuno H, Namiki N, Sasaki T, Katayose Y, Matsumoto T, Wu J (2013). A BAC physical map of aus rice cultivar ‘Kasalath’, and the map-based genomic sequence of ‘Kasalath’ chromosome 1. Plant J.

[CR24] Kawahara Y, Oono Y, Kanamori H, Matsumoto T, Itoh T, Minami E (2012). Simultaneous RNA-seq analysis of a mixed transcriptome of rice and blast fungus interaction. PLoS One.

[CR25] Kawahara Y, de la Bastide M, Hamilton J, Kanamori H, McCombie R, Ouyang S (2013). Improvement of the *Oryza sativa* Nipponbare reference genome using next generation sequence and optical map data. Rice.

[CR26] Kawahara Y, Oono Y, Wakimoto H, Ogata J, Kanamori H, Sasaki H, Mori S, Matsumoto T, Itoh T (2016). TENOR: Database for Comprehensive mRNA-Seq Experiments in Rice. Plant Cell Physiol.

[CR27] Kikuchi S, Satoh K, Nagata T, Kawagashira N, Doi K, Kishimoto N (2003). Collection, mapping, and annotation of over 28,000 cDNA clones from *japonica* rice. Science.

[CR28] Kojima S, Takahashi Y, Kobayashi Y, Monna L, Sasaki T, Araki T, Yano M (2002). Hd3a, a rice ortholog of the Arabidopsis FT gene, promotes transition to flowering downstream of Hd1 under short-day conditions. Plant Cell Physiol.

[CR29] Kojima Y, Ebana K, Fukuoka S, Nagamine T, Kawase M (2005). Development of an RFLP-based rice diversity research set of germplasm. Breeding Sci.

[CR30] Komiya R, Ikegami A, Tamaki S, Yokoi S, Shimamoto K (2008). *Hd3a* and *RFT1* are essential for flowering in rice. Development.

[CR31] Konishi S, Izawa T, Lin SY, Ebana K, Fukuta Y, Sasaki T, Yano M (2006). An SNP caused loss of seed shattering during rice domestication. Science.

[CR32] Kumagai M, Kim J, Itoh R, Itoh T (2013). Tasuke: a web-based visualization program for large-scale resequencing data. Bioinformatics.

[CR33] Kurata N, Yamazaki Y (2006). Oryzabase. An integrated biological and genome information database for rice. Plant Physiol.

[CR34] Matsubara K, Yamanouchi U, Nonoue Y, Sugimoto K, Wang ZX, Minobe Y, Yano M (2011). *Ehd3*, encoding a plant homeodomain finger-containing protein, is a critical promoter of rice flowering. Plant J.

[CR35] Matsubara K, Ogiso-Tanaka E, Hori K, Ebana K, Ando T, Yano M (2012). Natural variation in *Hd17*, a homolog of Arabidopsis *ELF3* that is involved in rice photoperiodic flowering. Plant Cell Physiol.

[CR36] Mizuno H, Wu J, Kanamori H, Fujisawa M, Namiki N, Saji S, Katagiri S, Katayose Y, Sasaki T, Matsumoto T (2006). Sequencing and characterization of telomere and subtelomere regions on rice chromosomes 1S, 2S, 2L, 6L, 7S, 7L and 8S. Plant J.

[CR37] Mizuno H, Wu J, Katayose Y, Kanamori H, Sasaki T, Matsumoto T (2008). Characterization of chromosome ends on the basis of the structure of TrsA subtelomeric repeats in rice (*Oryza sativa* L.). Mol Genet Genomics.

[CR38] Mizuno H, Wu J, Katayose Y, Kanamori H, Sasaki T, Matsumoto T (2008). Chromosome-specific distribution of nucleotide substitutions in telomeric repeats of rice (*Oryza sativa* L.). Mol Biol Evol.

[CR39] Mizuno H, Kawahara Y, Sakai H, Kanamori H, Wakimoto H, Yamagata H, Oono Y, Wu J, Ikawa H, Itoh T, Matsumoto T (2010). Massive parallel sequencing of mRNA in identification of unannotated salinity, stress-inducible transcripts in rice (*Oryza sativa* L.). BMC Genomics.

[CR40] Mizuno H, Kawahara Y, Wu J, Katayose Y, Kanamori H, Ikawa H, Itoh T, Sasaki T, Matsumoto T (2011). Asymmetric distribution of gene expression in the centromeric region of rice chromosome 5. Front Plant Sci.

[CR41] Mizuno H, Wu J, Matsumoto T (2014). Characterization of chromosomal ends on the basis of chromosome-specific telomere variants and subtelomeric repeats in rice (*Oryza sativa* L.). Subtelomeres.

[CR42] Nagaki K, Cheng Z, Ouyang S, Talbert PB, Kim M, Jones KM, Henikoff S, Buell CR, Jiang J (2004). Sequencing of a rice centromere uncovers active genes. Nat Genet.

[CR43] Nagano AJ, Sato Y, Mihara M, Antonio BA, Motoyama R, Itoh H, Nagamura Y, Izawa T (2012). Deciphering and prediction of transcriptome dynamics under fluctuating field conditions. Cell.

[CR44] Ogiso-Tanaka E, Matsubara K, Yamamoto S, Nonoue Y, Wu J, Fujisawa H, Ishikubo H, Tanaka T, Ando T, Matsumoto T, Yano M (2013). Natural variation of the *RICE FLOWERING LOCUS T 1* contributes to flowering time divergence in rice. PLoS One.

[CR45] Oikawa T, Maeda H, Oguchi T, Yamaguchi T, Tanabe N, Ebana K, Yano M, Ebitani T, Izawa T (2015). The birth of a black rice gene and its spread by introgression. Plant Cell.

[CR46] Okazaki Y, Saito K (2016). Integrated metabolomics and phytochemical genomics approaches for studies in rice. GigaScience.

[CR47] Oono Y, Kawahara Y, Kanamori H, Mizuno H, Yamagata H, Yamamoto M, Hosokawa S, Ikawa H, Akahane I, Zhu Z, Wu J, Itoh T, Matsumoto T (2011). mRNA-seq reveals a comprehensive transcriptome profile of rice under phosphate stress. Rice.

[CR48] Oono Y, Kawahara Y, Yazawa T, Kanamori H, Kuramata M, Yamagata H, Hosokawa S, Minami H, Ishikawa S, Wu J, Antonio B, Handa H, Itoh T, Matsumoto T (2013). Diversity in the complexity of phosphate starvation transcriptomes among rice cultivars based on RNA-Seq profiles. Plant Mol Biol.

[CR49] Oono Y, Yazawa T, Kawahara Y, Kanamori H, Kobayashi F, Sasaki H, Mori S, Wu J, Handa H, Itoh T, Matsumoto T (2014). Genome-wide transcriptome analysis reveals that cadmium stress signaling controls the expression of genes in drought stress signal pathways in rice. PLoS One.

[CR50] Paterson A, Bowers J, Bruggmann R, Dubchak I, Grimwood J, Gundlach H (2009). The *Sorghum bicolor* genome and the diversification of grasses. Nature.

[CR51] Rice Annotation Project (2008). The Rice Annotation Project Database (RAP-DB): 2008 update. Nucleic Acids Res.

[CR52] Sakai H, Ikawa H, Tanaka T, Numa H, Minami H, Fujisawa M, Shibata M, Kurita K, Kikuta A, Hamada M, Kanamori H, Namiki N, Wu J, Itoh T, Matsumoto T, Sasaki T (2011). Distinct evolutionary patterns of *Oryza glaberrima* deciphered by genome sequencing and comparative analysis. Plant J.

[CR53] Sakai H, Lee SS, Tanaka T, Numa H, Kim J, Kawahara Y, Wakimoto H, Yang CC, Iwamoto M, Abe T, Yamada Y, Muto A, Inokuchi H, Ikemura T, Matsumoto T, Sasaki T, Itoh T (2013). Rice Annotation Project Database (RAP-DB): an integrative and interactive database for rice genomics. Plant Cell Physiol.

[CR54] Sakai H, Kanamori H, Arai-Kichise Y, Shibata-Hatta M, Ebana K, Oono Y, Kurita K, Fujisawa H, Katagiri S, Mukai Y, Hamada M, Itoh T, Matsumoto T, Katayose Y, Wakasa K, Yano M, Wu J (2014). Construction of pseudomolecule sequences of the aus rice cultivar Kasalath for comparative genomics of Asian cultivated rice. DNA Res.

[CR55] Sato Y, Antonio B, Namiki N, Motoyama R, Sugimoto K, Takehisa H, Minami H, Kamatsuki K, Kusaba M, Hirochika H, Nagamura Y (2011). Field transcriptome revealed critical developmental and physiological transitions involved in the expression of growth potential in *japonica* rice. BMC Plant Biol.

[CR56] Schmutz J, Cannon SB, Jessica Schlueter J, Ma J, Mitros T, Nelson W, Hyten DL (2010). Genome sequence of the palaeopolyploid soybean. Nature.

[CR57] Schnable P, Ware D, Fulton RS, Joshua C, Stein JC, Fusheng Wei F, Shiran Pasternak S (2009). The B73 maize genome: complexity, diversity, and dynamics. Science.

[CR58] Shimono M, Sugano S, Nakayama A, Jiang CJ, Ono K, Toki S, Takatsuji H (2007). Rice WRKY45 plays a crucial role in benzothiadiazole-inducible blast resistance. Plant Cell.

[CR59] Shomura A, Izawa T, Ebana K, Ebitani T, Kanegae H, Konishi S, Yano M (2008). Deletion in a gene associated with grain size increased yields during rice domestication. Nat Genet.

[CR60] Sugimoto K, Takeuchi Y, Ebana K, Miyao A, Hirochika H, Hara N, Ishiyama K, Kobayashi M, Ban Y, Hattori T, Yano M (2010). Molecular cloning of *Sdr4*, a regulator involved in seed dormancy and domestication of rice. Proc Natl Acad Sci U S A.

[CR61] Taguchi-Shiobara F, Kawagoe Y, Kato H, Onodera H, Tagiri A, Hara N, Miyao A, Hirochika H, Kitano H, Yano M, Toki S (2011). A loss-of-function mutation of rice *DENSE PANICLE 1* causes semi-dwarfness and slightly increased number of spikelets. Breeding Sci.

[CR62] Takagi K, Ishikawa N, Maekawa M, Tsugane K, Iida S (2007). Transposon display for active DNA transposons in rice. Genes Genet Syst.

[CR63] Takahashi Y, Shomura A, Sasaki T, Yano M (2001). Hd6, a rice quantitative trait locus involved in photoperiod sensitivity, encodes the alpha subunit of protein kinase CK2. Proc Natl Acad Sci U S A.

[CR64] Takahashi Y, Teshima KM, Yokoi S, Innan H, Shimamoto K (2009). Variations in Hd1 proteins, Hd3a promoters, and Ehd1 expression levels contribute to diversity of flowering time in cultivated rice. Proc Natl Acad Sci U S A.

[CR65] Takai T, Adachi S, Taguchi-Shiobara F, Sanoh-Arai Y, Iwasawa N, Yoshinaga S (2013). A natural variant of *NAL1*, selected in high-yield rice breeding programs, pleiotropically increases photosynthesis rate. Sci Rep.

[CR66] The 3,000 Rice Genomes Project (2014). The 3,000 rice genomes project. GigaScience.

[CR67] Ueguchi-Tanaka M, Ashikari M, Nakajima M, Itoh H, Katoh E, Kobayashi M, Chow TY, Hsing YI, Kitano H, Yamaguchi I, Matsuoka M (2005). GIBBERELLIN INSENSITIVE DWARF1 encodes a soluble receptor for gibberellin. Nature.

[CR68] Uga Y, Sugimoto K, Ogawa S, Rane J, Ishitani M, Hara N, Kitomi Y, Inukai Y, Ono K, Kanno N, Inoue H, Takehisa H, Motoyama R, Nagamura Y, Wu J, Matsumoto T, Takai T, Okuno K, Yano M (2013). Control of root system architecture by *DEEPER ROOTING 1* increases rice yield under drought conditions. Nat Genet.

[CR69] Wang ZX, Yano M, Yamanouchi U, Iwamoto M, Monna L, Hayasaka H, Katayose Y, Sasaki T (1999). The Pib gene for rice blast resistance belongs to the nucleotide binding and leucine-rich repeat class of plant disease resistance genes. Plant J.

[CR70] Wang M, Yu Y, Haberer G, Marri PR, Fan C, Goicoechea JL, Zuccolo A, Song X, Kudrna D, Ammiraju JS, Cossu RM, Maldonado C, Chen J, Lee S, Sisneros N, Baynast K, Golser W, Wissotski M, Kim W, Sanchez P, Ndjiondjop MN, Sanni K, Long M, Carney J, Panaud O, Wicker T, Machado CA, Chen M, Mayer KF, Rounsley S, Wing RA (2014). The genome sequence of African rice (*Oryza glaberrima*) and evidence for independent domestication. Nat Genet.

[CR71] Wei FJ, Droc G, Guiderdoni E, Hsing YI (2013). International Consortium of Rice Mutagenesis: resources and beyond. Rice.

[CR72] Wing RA, Kim H, Goicoechea JL, Yu Y, Kudrna D, Zuccolo A, Ammiraju J, Luo M, Nelson W, Ma J, Sanmiguel P, Hurwitz B, Ware D, Brar D, Mackill D, Soderlund C, Stein L, Jackson S (2007). The oryza map alignment project (OMAP): A new resource for comparative genome studies within oryza. Rice Functional Genomics: Challenges, Progress and Prospects.

[CR73] Wu J, Yamagata H, Hayashi-Tsugane M, Hijishita S, Fujisawa M, Shibata M, Ito Y, Nakamura M, Sakaguchi M, Yoshihara R, Kobayashi H, Ito K, Karasawa W, Yamamoto M, Saji S, Katagiri S, Kanamori H, Namiki N, Katayose Y, Matsumoto T, Sasaki T (2004). Composition and structure of the centromeric region of rice chromosome 8. Plant Cell.

[CR74] Wu J, Mizuno H, Sasaki T, Matsumoto T (2008). Comparative analysis of rice genome sequence to understand the molecular basis of genome evolution. Rice.

[CR75] Wu J, Fujisawa M, Tian Z, Yamagata H, Kamiya K, Shibata M, Hosokawa S, Ito Y, Hamada M, Katagiri S, Kurita K, Yamamoto M, Kikuta A, Machita K, Karasawa W, Kanamori H, Namiki N, Mizuno H, Ma J, Sasaki T, Matsumoto T (2009). Comparative analysis of complete orthologous centromeres from two subspecies of rice reveals rapid variation of centromere organization and structure. Plant J.

[CR76] Yamamoto T, Nagasaki H, Yonemaru J, Ebana K, Nakajima M, Shibaya T, Yano M (2010). Fine definition of the pedigree haplotypes of closely related rice cultivars by means of genome-wide discovery of single-nucleotide polymorphisms. BMC Genomics.

[CR77] Yamane H, Ito T, Ishikubo H, Fujisawa M, Yamagata H, Kamiya K, Ito Y, Hamada M, Kanamori H, Ikawa H, Katayose Y, Wu J, Sasaki T, Matsumoto T (2009). Molecular and evolutionary analysis of the *Hd6* photoperiod sensitivity gene within genus Oryza. Rice.

[CR78] Yan H, Ito H, Nobuta K, Ouyang S, Jin W, Tian S, Lu C, Venu RC, Wang GL, Green PJ, Wing RA, Buell CR, Meyers BC, Jiang J (2006). Genomic and genetic characterization of rice Cen3 reveals extensive transcription and evolutionary implications of a complex centromere. Plant Cell.

[CR79] Yang CC, Kawahara Y, Mizuno H, Wu J, Matsumoto T, Itoh T (2012). Independent domestication of Asian rice followed by gene flow from *japonica* to *indica*. Mol Biol Evol.

[CR80] Yang W, Duan L, Chen G, Xiong L, Liu Q (2013). Plant phenomics and high-throughput phenotyping: accelerating rice functional genomics using multidisciplinary technologies. Curr Opin Plant Biol.

[CR81] Yano M, Kojima S, Takahashi Y, Lin H, Sasaki T (2001). Genetic control of flowering time in rice, a short-day plant. Plant Physiol.

[CR82] Zhang Y, Huang Y, Zhang L, Li Y, Lu T, Lu Y, Feng Q, Zhao Q, Cheng Z, Xue Y, Wing RA, Han B (2004). Structural features of the rice chromosome 4 centromere. Nucleic Acids Res.

[CR83] Zhou S, Bechner MC, Place M, Churas CP, Pape L, Leong SA, Runnheim R, Forrest DK, Goldstein S, Livny M, Schwartz DC (2007). Validation of rice genome sequence by optical mapping. BMC Genomics.

